# Medical Appointment—Prof. Rauch

**Published:** 1857-08

**Authors:** 


☞ We see in the July number of the Northwestern Medical and Surgical Journal, that John H. Rauch, M. D., of Burlington, Iowa, has received an appointment to the chair of Materia Medica, in the Rush Medical College. We congratulate the Faculty of that school upon the acquisition, for we believe Dr. R. eminently qualified to fill the position, and that in him they have gained a new element of strength and usefulness. Dr. R. has already, in a private position, proven himself to be an ardent student and accomplished scholar in the department assigned him, and from personal acquaintance, we feel assured he will prove himself an efficient teacher of that knowledge of which he is so thoroughly the master.

From the same source where we find notice of the above appointment, we learn that our old and much esteemed friend Prof. H. A. Johnson, the former incumbent of the chair to which Dr. R. has been appointed, is transferred to the chair of Physiology, made vacant by the resignation of Prof. Herrick; and Dr. W. H. Byeford, of Evansville, Ia., has been appointed to the chair of Obstetrics, &c., made vacant by resignation of Prof. Evans. W. R. M.

